# Aspirin Eugenol Ester Attenuates Paraquat-Induced Hepatotoxicity by Inhibiting Oxidative Stress

**DOI:** 10.3389/fphys.2020.582801

**Published:** 2020-10-22

**Authors:** Zhen-Dong Zhang, Mei-Zhou Huang, Ya-Jun Yang, Xi-Wang Liu, Zhe Qin, Shi-Hong Li, Jian-Yong Li

**Affiliations:** Key Lab of New Animal Drug Project of Gansu Province, Key Lab of Veterinary Pharmaceutical Development of Ministry of Agriculture and Rural Affairs, Lanzhou Institute of Husbandry and Pharmaceutical Sciences of CAAS, Lanzhou, China

**Keywords:** aspirin eugenol ester, paraquat, oxidative stress, hepatotoxicity, reactive oxygen species

## Abstract

Aspirin eugenol ester (AEE) is a new potential drug with anti-inflammatory and antioxidant stress pharmacological activity. Paraquat (PQ) is an effective and commercially important herbicide that is widely used worldwide. However, paraquat is highly toxic and can cause various complications and acute organ damage, such as liver, kidney and lung damage. The purpose of this study was to investigate whether AEE has a protective effect on hepatotoxicity induced by PQ *in vivo* and *in vitro*. Cell viability, apoptosis rate, mitochondrial function and intracellular oxidative stress were detected to evaluate the protective effect of AEE on PQ-induced BRL-3A (normal rat hepatocytes) cytotoxicity *in vitro*. *In vivo*, AEE pretreatment could attenuate oxidative stress and histopathological changes in rat liver induced by PQ. The results showed that AEE could reduce the hepatotoxicity induced by PQ *in vivo* and *in vitro*. AEE reduced PQ-induced hepatotoxicity by inhibitingoxidative stress and maintaining mitochondrial function. This study proved that AEE is an effective antioxidant and can reduce the hepatotoxicity of PQ.

## Graphical Abstract

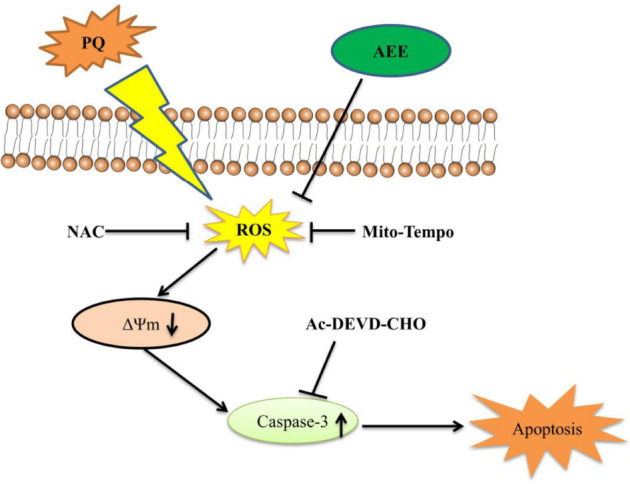

## Introduction

Paraquat (PQ) is a non-selective herbicide with excellent effect, which has been widely used in the world for many years (Sun et al., 2014; Chen H.G. et al., 2019; Shao et al., 2019). PQ is extremely toxic to humans (Xu et al., 2017; Chen F. et al., 2019). Studies have shown that when ingesting about 10 ml PQ, patients can die of multiple organ failure a few hours later (Feng et al., 2018). The accumulation of PQ can damage the main organs such as lung, kidney, liver and heart (Yan et al., 2017). It is reported that the liver is one of the main target organs of PQ poisoning, which is often accompanied by the formation of free radicals (Han et al., 2014; El-Boghdady et al., 2017). The liver is the main metabolic and detoxifying organ of the human body (Jung and Kim, 2018; Zhang et al., 2018). It is well known that drugs and other substances are further transformed and metabolized after being absorbed by the body, resulting in the production of free radicals in the liver. Excessive free radicals produce oxidative stress on the liver, which in turn leads to oxidative damage to the liver (Parvez et al., 2018).

Currently, the molecular mechanism of hepatotoxicity induced by PQ is not completely understood. It is known that the redox response is one of the main factors involved in the toxic effects of PQ (Ren et al., 2019). It has been reported that PQ molecules can interfere with the electron transport chain and then inhibit the synthesis of NADPH (Gao et al., 2018). Excessive production of reactive oxygen species (ROS) was observed during PQ poisoning, indicating that oxidative stress was involved in the pathological changes induced by PQ. Excessive ROS and excessive free radicals lead to oxidative stress by destroying DNA, proteins and lipids (Qian et al., 2019). Therefore, the premise of the toxic effect of PQ is its induced oxidative stress. At present, the main methods for the treatment of PQ poisoning are immunosuppressant and hemodialysis. Existing clinical treatments for severe PQ poisoning only relieve symptoms (Gawarammana and Buckley, 2011). In recent decades, new drugs to treat the toxicity of PQ have been developed. In the early stages of poisoning, the use of antioxidants has been shown to effectively reduce the damage of PQ to organs. Therefore, it is imperative to develop potential effective drugs for the treatment of PQ poisoning.

Aspirin eugenol ester (AEE) is a new potential pharmaceutical compound possessing anti-inflammatory and anti-oxidative stress pharmacological activity (Li J.Y. et al., 2011; Li J.Y. et al., 2012; Ma et al., 2015; Ma et al., 2017; Huang et al., 2019). The effect of AEE against H_2_O_2_-induced oxidative stress of human umbilical vein endothelial cells (HUVECs) is content with AEE-enhanced the expression of Bcl2 and Nrf2 (Huang et al., 2019). It has been well documented that AEE could alleviate H_2_O_2_-induced dysfunction of mitochondria, the generation of ROS production and the increase of apoptosis via enhancing the expression of Bcl2 and Nrf2 (Huang et al., 2019). It is well known that the dysfunction of mitochondria could release cytochrome C, apoptosis inducing factor (AIF), and other factor into cytoplasm to mediate downstream apoptotic signals causing cell apoptosis (Abate et al., 2019b; Hasnat et al., 2019), while the exacerbation of reactive oxygen species (ROS) induced by the dysfunction of mitochondria is also vital incentive of cell apoptosis (Meng et al., 2018; Moradzadeh et al., 2018). The determination of whether AEE could attenuate PQ-induced hepatotoxicity by inhibiting oxidative stress requires further study.

In this study, we used normal rat hepatocytes (BRL-3A cells) and male sprague-dawley rats to investigate whether AEE can reduce PQ-induced hepatotoxicity and to explore the possible protective mechanism of AEE. The results showed that AEE could inhibit oxidative stress and reduce the hepatotoxicity induced by PQ.

## Materials and Methods

### Chemicals

Aspirin eugenol ester (99.5%) was prepared in Lanzhou Institute of Husbandry and Pharmaceutical Sciences of CAAS (Lanzhou, China). BRL-3A cells were purchased from Cell Bank of the typical Culture Preservation Committee of the Chinese Academy of Sciences (Shanghai, China). Methyl viologen dichloride was purchased from Aladdin (Shanghai, China). Dimethyl sulfoxide (DMSO) was supplied by Sigma (St. Louis, MO). 0.05%Trypsin-EDTA, DMEM, and fetal bovine serum was from Gibco (Grand Island, NY, United States). Cell counting kit-8, GSH and GSSG assay kit, total Glutathione peroxidase assay kit, caspase-3 activity assay kit, enhanced ATP assay kit, reactive oxygen species (ROS) assay kit, DCFH-DA kit, DHE kit, superoxide dismutase (SOD), and malondialdehyde (MDA) assay kit were purchased from Beyotime (Shanghai, China). Anti-Bcl-2 (1:1000), Anti-Bax (1:5000), Anti-caspase-3 (1:500), β-actin (1:2000) and goat anti-rabbit IgG (1:5000) were purchased from abcam (Shanghai, China). BCA protein assay kit was supplied by Solarbio (Beijing, China). Mito Tracker Red CMXRos probe and MitoSOX Red Mitochondrial Superoxide Indicator were from Thermo Scientific (Waltham, MA, United States). Alanine aminotransferase (ALT) kit and aspartate aminotransferase (AST) kit were purchased from Mlbio (Shanghai, China). An Annexin V/FITC apoptosis detection kit was from BD Biosciences (San Diego, CA, United States).

### Animal Experiment

Thirty male specific pathogen-free SD rats (6 weeks old) weighing 120–130 g were purchased from the Laboratory Animal Center of Lanzhou Veterinary Research Institute (Lanzhou, China). All animals were housed in groups in SPF-class housing of laboratory at a controlled relative humidity (55–65%), 12 h light/dark cycle and temperature (24 ± 2°C). The rats were randomly assigned into five groups (*n* = 6): (1) control group, in which rats were administrated equivalent saline by intraperitoneal injection (ip); (2) PQ group, in which rats were administrated PQ (20 mg/kg body weight, ip); (3–5) Three AEE groups, in which rats were pre-administrated AEE (27, 54, 108 mg/kg/day body weight, respectively) by gavage once a day for 1 weeks before being administrated PQ. The rats in the different groups were sacrificed after a single intraperitoneal injection of 20 mg/kg PQ for 24 h. All experimental protocols and procedures were approved by the Institutional Animal Care and Use Committee of Lanzhou Institute of Husbandry and Pharmaceutical Science of Chinese Academy of Agricultural Sciences (Approval No. NKMYD201907018; Approval Date: 18 July 2019). Animal welfare and experimental procedures were performed strictly in accordance with the Guidelines for the Care and Use of Laboratory Animals issued by the United States National Institutes of Health.

### Serum Biochemistry Analysis

Blood samples were collected from rats into EDTA-containing tubes. Serum samples were obtained by standing at room temperature for 30 min and centrifuging blood at 4000 rpm for 10 min at 4°C and then stored at −80°C until analysis. Serum alanine aminotransferase (ALT) and aspartate aminotransferase (AST) activity was determined using a commercial kit (Mlbio, Shanghai, China).

### Histological Examination

Liver specimens were fixed with 10% formaldehyde. After fixation, the liver tissue was embedded in paraffin wax, sectioned to a thickness of 5 μm and stained with hematoxylin-eosin staining (H&E).

### Cell Culture and Treatments

BRL-3A cells were maintained in DMEM medium supplemented with 10% (*v/v*) fetal bovine serum and antibiotics (100 μg/mL penicillin and 100 μg/mL streptomycin) in a humidified atmosphere with 5% CO_2_ at 37°C.

### Cell Viability Assay

BRL-3A cell viability was determined using the Cell Counting Kit-8 (CCK-8). Briefly, 3 × 10^5^ cells were seeded in 96-well plates. After treatment, 10 μL CCK-8 reagent was added to each well. Cells were incubated for 2 h at 37°C in a humidified 5% CO_2_ atmosphere. Finally, cell viability was determined by measuring the absorbance at 480 nm using a microplate reader (Multiskan^TM^ FC; Thermo Scientific^TM^, United States).

### Measurement of Intracellular Superoxide Anion and Total Intracellular and Mitochondrial ROS Generation

The MitoSOX^TM^ red mitochondrial superoxide indicator, reactive oxygen species assay kit and dihydroethidium (DHE) were used to detect mitochondrial ROS, total intracellular ROS and superoxide anion, respectively. Briefly, cells were cultured on glass coverslips in 24-well culture plates. Cells were incubated with red mitochondrial superoxide indicator, DCFH-DA probes and DHE for 30 min at 37°C in the dark. The fluorescence intensity in BRL-3A cells was detected.

### Measurement of Mitochondrial Membrane Potential (ΔΨm)

The Mito Tracker Red CMXRos probe was used to detect ΔΨm. Briefly, BRL-3A cells were cultured and processed in 24-well glass bottom cell culture plates. The cells were incubated with phenol red-free medium containing the MitoTracker Red CMXRos probe (10 μM) at 37°C in the dark for 25 min. Confocal images were captured using a Zeiss confocal laser scanning microscope (LSM 800, Zeiss, Germany).

### Measurement of ATP Levels

After different treatments, the intracellular ATP level of each group was measured. The ATP levels were quantified using an enhanced ATP assay kit. Solutions were prepared according to manufacturer instructions. The fluorescence intensity in BRL-3A cells was detected by Multimode plate reader (Enspire, PerkinElmer, United States).

### Analysis of MDA, SOD, Caspase-3, GSH/GSSH and GPx

The levels of MDA, GSH/GSSH ratio, SOD and the activity of caspase-3 and GPx in BRL-3A cells and in the rat liver were assessed using the corresponding commercial kits according to the manufacturer’s protocols.

### Flow Cytometric Analysis

Cells (1 × 10^5^ cells per well) were seeded in a six-well microplate. After different treatments as indicated, the cells were collected and washed with PBS. 5 μL of Annexin V and 5 μL of 7-amino-actinomycin (7-AAD) were added in 100 μL suspended cells, and the mixture was incubated at room temperature for 25 min. All of the samples were analyzed immediately by a flow cytometer (BD FACSVerse, United States).

### Protein Expression Analysis

The expression of caspase-3, Bax and Bcl-2 among different treatment for BRL-3A was assessed by Western blot analysis. In brief, total protein of the cells was extracted using RIPA, quantified by bicinchoninic acid (BCA) method, and separated by precast SDS-PAGE Gel (15%, 4–20%). The separated proteins were transferred onto polyvinylidene fluoride (PVDF) membrane using standard procedures. Blots were incubated with the primary antibody (Anti-Bcl-2 (1:1000), Anti-Bax (1:5000) and Anti-caspase-3 (1:500), Anti-β-actin (1:3000), Abcam, Shanghai, China) followed by horseradish peroxidase-conjugated secondary antibody (goat anti-rabbit IgG (1:5000), Abcam, Shanghai, China). Results were detected using the G: Box Chemi XRQ Imaging System (Cambridge, United Kingdom).

### Statistical Analysis

All experiments and data analysis were performed according the blinding principles (Altman et al., 1983; Huang et al., 2019). Statistical analyses were performed using SAS 9.2 (SAS Institute Inc., Cary, NC). All data are presented as means and standard deviations (SD). Differences between groups were analyzed by one-way analysis of variance (ANOVA). Statistical significance was considered at *p* < 0.05.

## Results

### AEE Administration Improves the Cell Viability of BRL-3A Cells Induced by PQ

**FIGURE 1:**
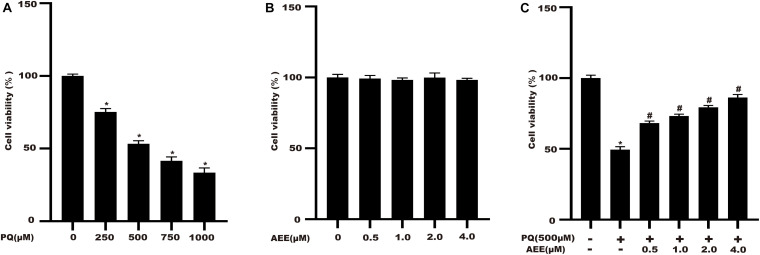
AEE administration improves the cell viability of BRL-3A cells induced by PQ. Cell viability was measured by CCK-8. **(A)** The effect of different concentrations of PQ on cell viability after 24 h. **(B)** The effect of different concentrations of AEE on cell viability after 24 h. **(C)** The effect of pretreatment with different AEE concentrations on cell viability in the presence of 500 μM PQ. Values are presented as the means ± SD where applicable (*n* = 6). *p < 0.05 compared with the control group; ^#^*p* < 0.05 compared with the PQ group.

BRL-3A cells were cultured with different concentrations of PQ (250, 500, 750, 1000 μM) for 24 h. The CCK-8 kit was used to evaluate the cytotoxic effect of PQ on BRL-3A cells. The results showed that cell viability decreased with increasing PQ concentration (Figure 1A). When the concentration reached 500 μM, cell viability decreased to about 50%. Therefore, 500 μM PQ was chosen as the optimal concentration for subsequent experiments. We also measured the cytotoxicity of AEE in BRL-3A cells. AEE alone did not affect cell viability (Figure 1B). Next, we investigated whether AEE could protect against PQ-induced cell viability. Cells were pretreated with various concentrations of AEE for 24 h and then stimulated with 500 μM PQ for 24 h. The results suggest that AEE has a strong protective effect against PQ-induced cell viability in a concentration-dependent manner (Figure 1C).

### AEE Antagonizes PQ-Induced Oxidative Stress in BRL-3A Cells

**FIGURE 2:**
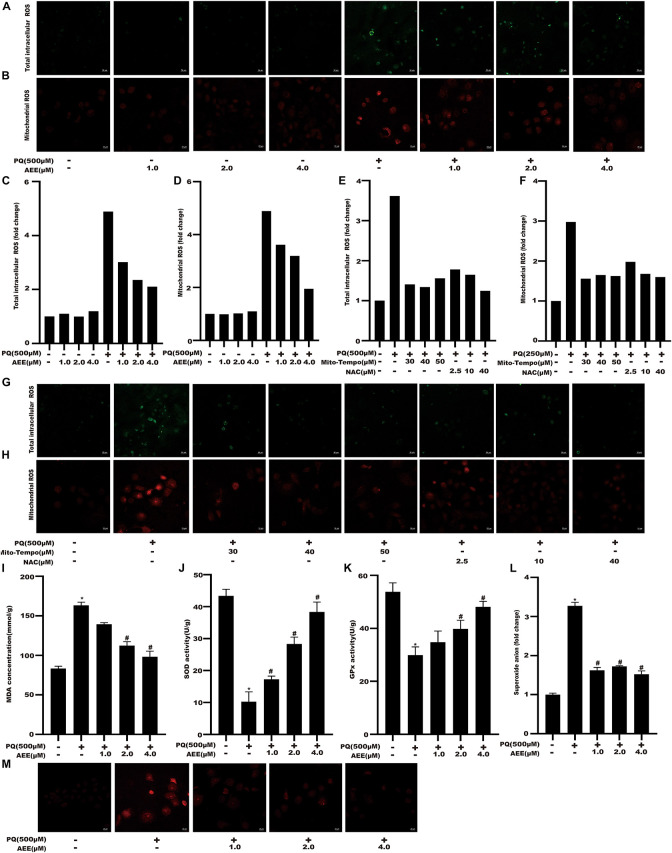
AEE reduces PQ-induced oxidative stress in BRL-3A cells. **(A,C)** Total intracellular ROS was detected in different treatment groups. Scale bar: 50 μm. **(B,D)** Mitochondrial ROS was detected in different treatment groups. Scale bar: 20 μm. **(E,G)** Total intracellular ROS was detected in different treatment groups. Scale bar: 50 μm. **(F,H)** Mitochondrial ROS was detected in different treatment groups. Scale bar: 20 μm. **(I)** MDA levels were detected in different treatment groups. **(J)** The activity of SOD in different treatment groups was detected. **(K)** The activity of GPx in different treatment groups was detected. **(L,M)** The level of superoxide anion was detected in different treatment groups. Scale bar: 20 μm. The bars display a mean of a number of replicates (*n* = 6). *p < 0.05 compared with the control group; ^#^*p* < 0.05 compared with the PQ group.

Previous studies have shown that PQ can cause cytotoxicity associated with increased ROS (Weimer et al., 2014; Koike et al., 2015; Zhang et al., 2018). Intracellular total ROS and mtROS levels were detected to validate changes in redox status of BRL-3A cells among different treatment groups. AEE alone did not change the total intracellular ROS and mtROS levels. Intracellular total ROS and mtROS levels of BRL-3A cells were significantly increased after exposure to 500 μM PQ for 24 h (Figures 2A–D). Mito-Tempo is a mitochondrial targeted antioxidant with the properties of scavenging superoxide and alkyl free radicals. N-acetylcysteine (NAC) is a scavenger of intracellular total ROS. We used Mito-Tempo and N-acetylcysteine (NAC), to verify the existence of intracellular ROS and mtROS production induced by PQ (Figures 2E–H). To elucidate the underlying mechanism of AEE in reducing PQ-induced BRL-3A cell damage, the level of MDA, superoxide anion and the activity of SOD, GPx were examined in various treatment groups of BRL-3A cells. Compared with the control, the level of MDA and superoxide anion were significantly increased and the activity of SOD and GPx were significantly decreased in the PQ treatment group. Moreover, SOD and GPx activity were significantly increased in the AEE pretreatment group, while the level of MDA and superoxide anion were significantly decreased (Figures 2I–M). The results showed that AEE could reduce the excessive production of ROS and superoxide anion induced by PQ.

### AEE Alleviates PQ-Induced Mitochondrial Dysfunction in BRL-3A Cells

**FIGURE 3:**
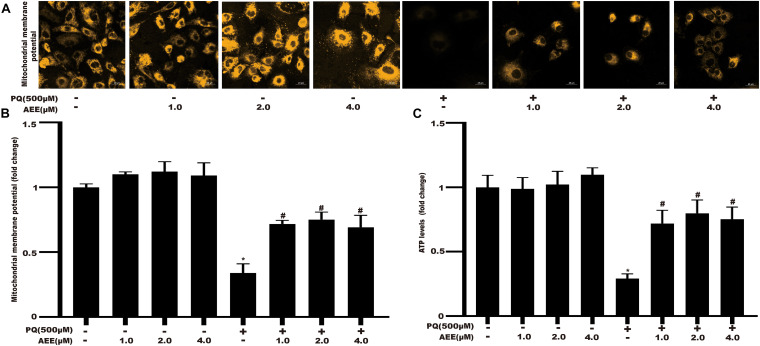
AEE alleviates PQ-induced mitochondrial dysfunction in BRL-3A cells. **(A,B)** The mitochondrial membrane potential (ΔΨm) of BRL-3A cells was measured in different treatment groups. Scale bar: 20 μm. **(C)** The ATP level of BRL-3A cells was measured in different treatment groups. Values are presented as the means ± SD where applicable (*n* = 6). *p < 0.05 compared with the control group; ^#^*p* < 0.05 compared with the PQ group.

Since AEE could significantly reduce the production of superoxide anion, we further studied the protective effect of AEE on mitochondrial dysfunction induced by PQ through the change of mitochondrial membrane potential (ΔΨm) and cellular ATP level. AEE alone did not affect the ΔΨm (Figure 3A). AEE pretreatment significantly inhibited the decrease of ΔΨm induced by PQ (Figure 3B). The sensitive index reflecting the function of mitochondria is the concentration of intracellular ATP (Liu et al., 2017; Kim et al., 2018). Similar to the previous results of ΔΨm, AEE alone did not affect the level of intracellular ATP, while AEE pretreatment significantly inhibited the decrease of intracellular ATP concentration induced by PQ (Figure 3C). These results suggested that AEE could significantly reduce PQ-induced mitochondrial dysfunction as a threshold effect at 1.0 μM by inhibiting intracellular oxidative stress, which are similar to previous study (Huang et al., 2019).

### AEE Mitigates PQ-Induced Apoptosis in BRL-3A Cells

**FIGURE 4:**
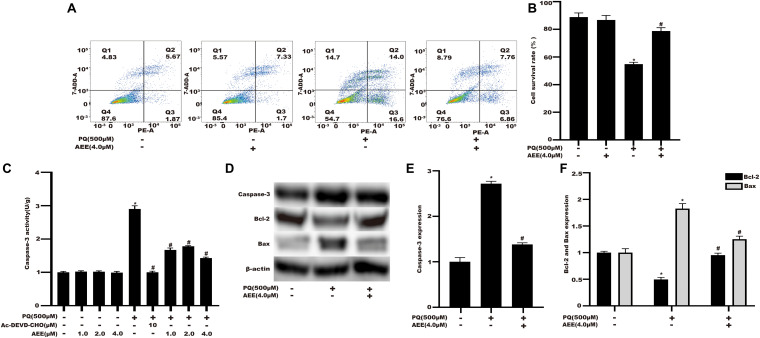
AEE mitigates PQ-induced apoptosis in BRL-3A cells. **(A)** The double stained with PE and 7-AAD results among different treatment groups. **(B)** The apoptosis rate among different treatment groups. **(C)** The caspase-3 activity of BRL-3A cells was determined in different treatment groups. **(D,E)** The expression of caspase-3 protein in BRL-3A cells was measured in different treatment groups. **(D,F)** The protein expression levels of Bcl-2 and Bax in BRL-3A cells were measured in different treatment groups. Values are presented as the means ± SD where applicable (*n* = 6). *p < 0.05 compared with the control group; ^#^*p* < 0.05 compared with the PQ group.

Studies have shown that apoptosis plays an important role in PQ-induced oxidative damage (Sagi and Kim, 2012). Next, we investigated whether the protective effect of AEE on PQ cytotoxicity occurs by inhibiting apoptosis. The results showed that the apoptosis rate of BRL-3A cells treated with PQ increased significantly (Figure 4A). Pretreatment with AEE significantly reduced the increase in apoptosis rate by PQ (Figure 4B). Cleaved caspase-3, a marker of apoptosis, also increased in PQ treatment group (Figure 4C). Pretreatment with different concentrations of AEE alone did not affect the activity of caspase-3. However, pretreatment with different concentrations of AEE could significantly inhibit the increase of caspase-3 activity induced by PQ (Figure 4C). Western blot analysis showed that compared with the control group, the expression of caspase-3 and Bax in BRL-3A cells treated with PQ was significantly up-regulated, while Bcl-2 was significantly down-regulated (Figures 4D–F). However, AEE pretreatment could inhibit the upregulation of active cleavage caspase-3 and Bax and the downregulation of Bcl-2 induced by PQ (Figures 4D–F). Overall, the results showed that AEE could inhibit PQ-induced apoptosis.

### AEE Reduces PQ-Induced Liver Injury in Rat

**FIGURE 5:**
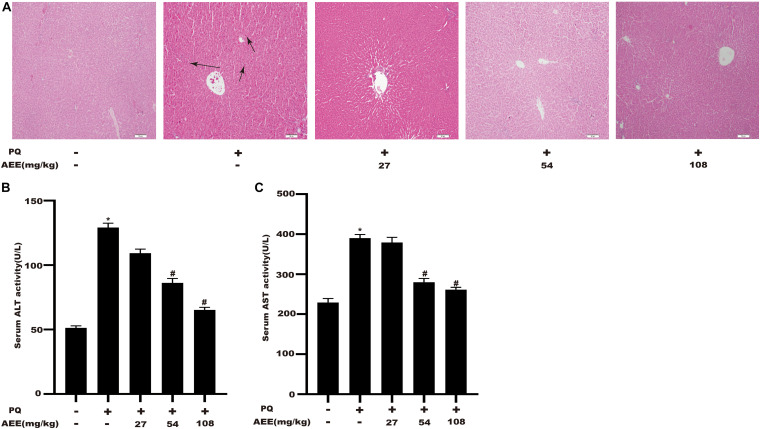
AEE reduces PQ-induced liver injury in rats. **(A)** Representative images of the morphological observations of histopathological changes. Scale bar: 100 μm. Note: The arrows of the pathological sections represent the cell atrophy and hyperemia. **(B)** The level of ALT in rat serum was measured in different treatment groups. **(C)** The level of AST in rat serum was measured in different treatment groups. Values are presented as the means ± SD where applicable (*n* = 6). *p < 0.05 compared with the control group; ^#^*p* < 0.05 compared with the PQ group.

To verify whether AEE has a protective effect on PQ-induced hepatotoxicity *in vivo*, we then explore the effect of AEE pretreatment on PQ-induced liver injury in rats. The results showed that a single intraperitoneal injection of 20 mg/kg PQ could cause obvious liver injury, which was characterized by a significant increase in serum AST and ALT (Figures 5B,C). AEE (54, 108 mg/kg) could significantly inhibit the increase of AST and ALT induced by PQ (Figures 5A,B). Furthermore, PQ (20 mg/kg) could significantly cause liver tissue necrosis, cell atrophy and portal hyperemia in rats. Pretreatment with AEE (54, 108 mg/kg) for seven consecutive days by gavage markedly attenuated reduce the pathological injury of liver tissue induced by PQ (Figure 5A). The results showed that AEE could effectively reduce the liver injury induced by PQ in rats.

### AEE Attenuates PQ-Induced Oxidative Stress in the Liver of Rat

**FIGURE 6:**
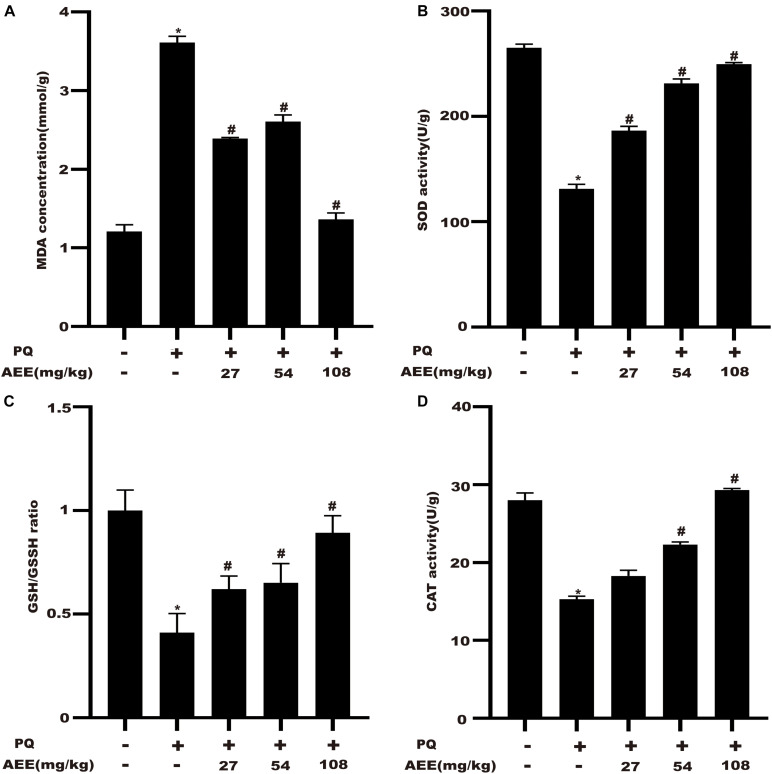
AEE attenuates PQ-induced hepatic oxidative stress in rats. **(A)** The level of MDA in rat liver was measured in different treatment groups. **(B)** The activity of SOD in rat liver was determined in different treatment groups. **(C)** The proportion of GSH/GSSH in rat liver was determined in different treatment groups. **(D)** The activity of CAT in rat liver was determined in different treatment groups. Values are presented as the means ± SD where applicable (*n* = 6). *p < 0.05 compared with the control group; ^#^*p* < 0.05 compared with the PQ group.

In order to investigate the inhibitory effect of AEE on liver oxidative stress, we detected the level of MDA, the activity of SOD and the ratio of GSH/GSSH. The results demonstrated that pretreatment with AEE (27, 54, 108 mg/kg) significantly suppressed the PQ-induced increase in MDA levels and the decrease in GSH/GSSH and SOD levels (Figures 6A–C). Compared with the control group, PQ treatment group could significantly reduce the activity of CAT. AEE pretreatment (54, 108 mg/kg) could significantly improve the activity of CAT (Figure 6D). These results suggested that AEE can effectively inhibit oxidative stress induced by PQ in rat liver.

## Discussion

On the basis of cell experiments *in vitro* and rat experiments *in vivo*, it was confirmed that AEE is an effective antioxidant and can reduce the hepatotoxicity induced by PQ. AEE attenuates PQ-induced hepatotoxicity by inhibiting the production of MDA, and intracellular superoxide anion.

Paraquat poisoning is caused by the selective accumulation of PQ molecules that can cause multiple organ failure and can cause severe damage to the liver (Qian et al., 2019). Although progress has been made in the comprehensive treatment of PQ poisoning, the mortality rate remains high due to the lack of effective treatment. The underlying mechanism of PQ poisoning has not been fully elucidated, but it may be multifactorial. Studies have shown that an important cause of PQ poisoning is the excessive production of ROS (Sun et al., 2011). The overproduction of reactive oxygen species could cause excessive oxidative stress and oxidant injury in cells (Kumar et al., 2016; Wang et al., 2017). In this work, we found that AEE increased the cell viability and the ΔΨm, decreased the activity of caspase-3, and inhibited the apoptosis of PQ-treated BRL-3A cells. In addition, our study found that AEE could reduce the superoxide anion induced by PQ in BRL-3A cells.

Aspirin eugenol ester is a new potential pharmaceutical compound possessing anti-inflammatory and anti-oxidative stress pharmacological activity (Li J. et al., 2012; Li J.Y. et al., 2012; Karam et al., 2015). The previous studies had certificated that AEE could reduce H_2_O_2_-induced mitochondrial dysfunction manifested as increased ROS and collapsed mitochondrial transmembrane potential by regulating Bcl2 and Nrf2 (Huang et al., 2019). Consistent with these previous studies, the present study also showed that AEE could ameliorate increased collapsed mitochondrial transmembrane potential induced by PQ. Moreover, the results also showed that AEE could enhance the antioxidant capacity of rats by increasing the level of SOD in serum and reduce ROS production in aorta induced by PQ. The present study coupled with the previous studies confirmed that the key factor of AEE alleviating hepatotoxicity induced by PQ is the inhibition of oxidative stress. It is mainly reflected in the inhibition of MDA production, superoxide anion production.

The purpose of this study was to investigate whether AEE can reduce hepatotoxicity induced by PQ. Previous studies have found that AEE inhibit the apoptosis induced by PQ *in vitro*. Some studies have shown that PQ-induced cytotoxicity is related to oxidative stress, including excessive production of ROS and lipid peroxidation (Matern et al., 2015; Ren et al., 2019; Schuttler et al., 2019). The main characteristics of oxidative stress are dysfunction of antioxidant system or excessive production of free radicals (El-Boghdady et al., 2017; Guo et al., 2017; Cavaliere et al., 2019; Lopes et al., 2019). Therefore, the common method to evaluate oxidative stress is to determine the level of superoxide anion and the changes of antioxidant system induced by ROS. The current results are consistent with those reported earlier, which proves that oxidative stress plays a key role in PQ-induced cytotoxicity. Mitochondria are the main source of ROS and also a target organelle for oxidative stress (Dodson et al., 2013; Zhu et al., 2014; Ito et al., 2015). The results of cellular oxidative stress showed that PQ induced mitochondrial dysfunction, which was characterized by the decrease of mitochondrial membrane potential, and the decrease of ATP production. One of the main consequences of cytotoxicity induced by PQ is apoptosis, which may also be the main mechanism of cell death induced by PQ. *In vivo* studies have also shown that PQ-induced hepatotoxicity is characterized by oxidative stress in rat liver, resulting in histopathological changes. Previous studies have found that AEE can significantly reduce PQ-induced hepatotoxicity *in vivo* and *in vitro*. AEE inhibited PQ-induced superoxide anion production, reduced lipid peroxidation, maintained mitochondrial function, and protected liver function, thereby inhibiting PQ-induced hepatotoxicity.

## Conclusion

In summary, the present study demonstrated that AEE is an effective antioxidant to reduce PQ-induced hepatotoxicity by inhibiting PQ-induced superoxide anion production, reducing lipid peroxidation and maintaining mitochondrial function.

## Data Availability Statement

The raw data supporting the conclusions of this article will be made available by the authors, without undue reservation.

## Ethics Statement

The animal study was reviewed and approved by the Institutional Animal Care and Use Committee of Lanzhou Institute of Husbandry and Pharmaceutical Science of Chinese Academy of Agricultural Sciences.

## Author Contributions

J-YL designed the experiments and wrote the manuscript. Z-DZ designed and performed the experiments and wrote the manuscript. M-ZH provided conceptualization and validation for the experiments and wrote the manuscript. Y-JY synthesized and purified AEE. S-HL, X-WL, and ZQ supplied reagents. All authors contributed to the article and approved the submitted version.

## Conflict of Interest

The authors declare that the research was conducted in the absence of any commercial or financial relationships that could be construed as a potential conflict of interest.

## Abbreviations

19m: mitochondrial membrane potential
AEE: aspirin eugenol ester
ALT: alanine transaminase
AST: aspartate aminotransferase
CAT: catalase
DMSO: dimethyl sulfoxide
GPx: glutathione peroxidase
GSH: reduced glutathione
GSSH: oxidized glutathione disulfide
HUVECs: human umbilical vein endothelial cells
Ip: intraperitoneal injection
mtROS: mitochondrial reactive oxygen species
MDA: malondialdehyde
NAC: N-acetylcysteine
NADPH: nicotinamide adenine dinucleotide phosphate
PQ: paraquat
PVDF: polyvinylidene fluoride
ROS: reactive oxygen species
SD: standard deviations
SOD: superoxide dismutase.
